# Supplementary material for: Tackling Intrinsic Antibiotic Resistance in Serratia marcescens with a Combination of Ampicillin/Sulbactam and Phage SALSA
Source: Antibiotics (Basel). 2020 Jul 1;9(7):371. doi: 10.3390/antibiotics9070371 (PMC7400198; doi:10.3390/antibiotics9070371)
Supplement: Supplementary file 1 [file antibiotics-09-00371-s001.zip › antibiotics-847709-supple-/Figure S2 ProteinComparison.pdf]

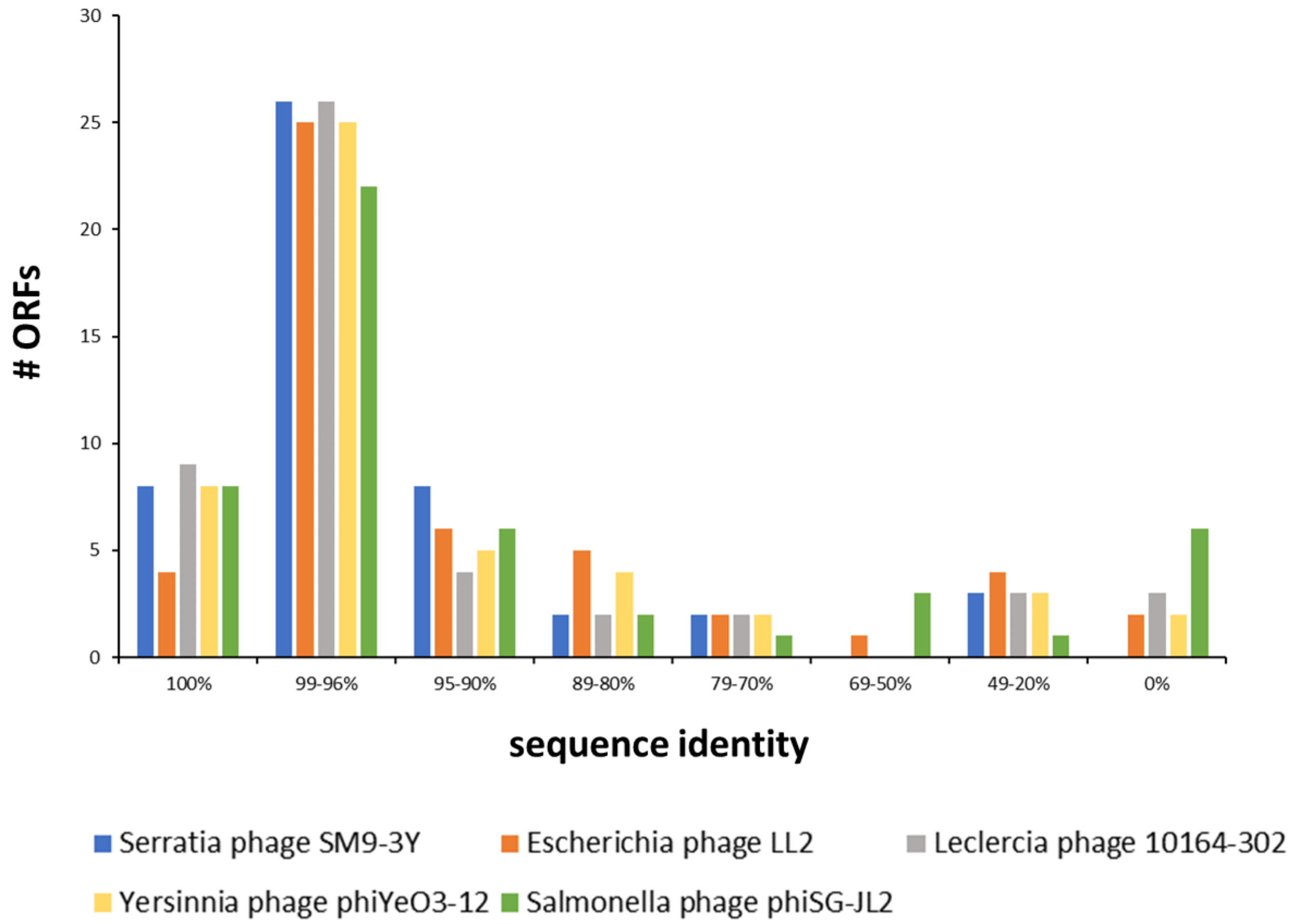

Supplementary Figure S2. Genome comparison at protein level. Sequence identity of translated ORFs of phage SALSA with five members of the T7-like viruses.
