# Supplementary material for: Tackling Intrinsic Antibiotic Resistance in Serratia marcescens with a Combination of Ampicillin/Sulbactam and Phage SALSA
Source: Antibiotics (Basel). 2020 Jul 1;9(7):371. doi: 10.3390/antibiotics9070371 (PMC7400198; doi:10.3390/antibiotics9070371)
Supplement: Supplementary file 1 [file antibiotics-09-00371-s001.zip › antibiotics-847709-supple-/FigureS1.pdf]

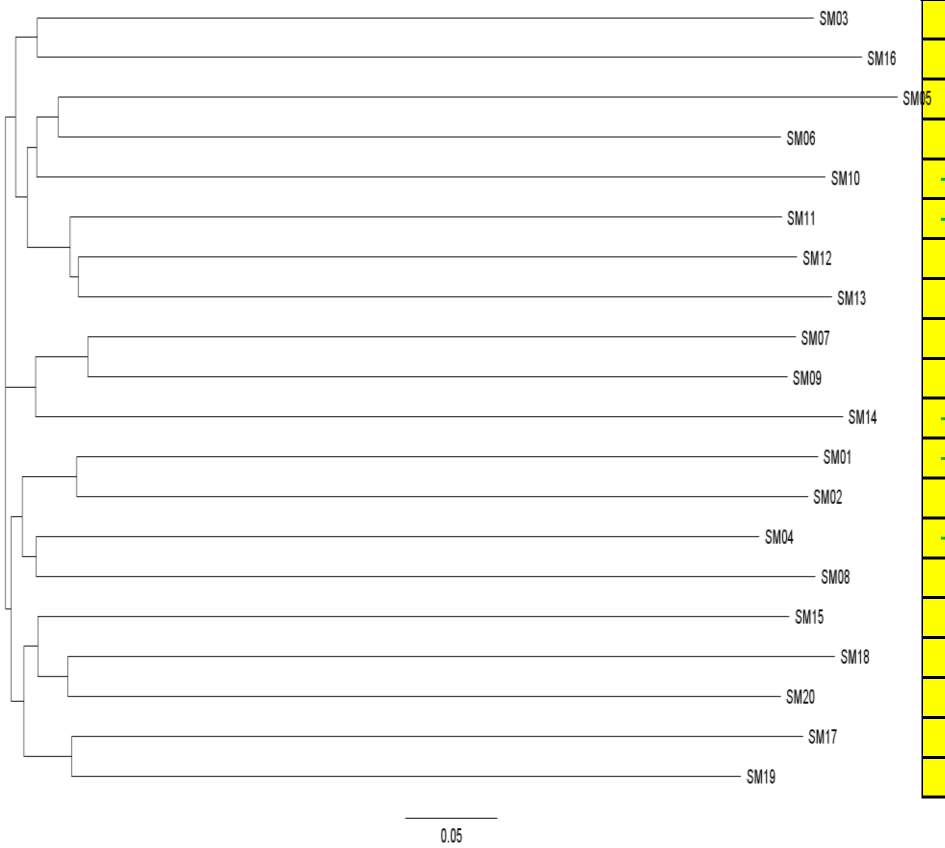

Phage Susceptibility	AMP	SAM	PIP	TZP	CTX	CPD	CXM	CXM axetil	CAZ	IPM	MEM	ETP	CIP	MXF	GEN	SXT	TGC
--	R	R	S	S	S	S	R	R	S	S	S	S	S	R	S	S	I
--	R	R	S	S	S	S	R	R	S	I	S	S	S	R	S	S	I
--	R	R	S	S	S	S	R	R	S	S	S	S	S	R	S	S	I
--	R	R	S	S	S	S	R	R	S	S	S	S	S	R	S	S	S
++	R	R	S	S	S	S	R	R	S	S	S	S	S	R	S	S	S
++	R	R	S	S	S	S	R	R	S	S	S	S	S	R	S	S	S
--	R	R	S	S	S	S	R	R	S	S	S	S	S	S	S	S	S
--	R	R	n.d.	S	S	R	R	R	S	S	S	n.d.	S	n.d.	S	S	S
--	R	R	S	S	S	R	R	R	S	S	S	S	S	R	S	S	S
--	R	R	S	S	S	S	R	R	S	S	S	S	S	R	S	S	S
++	R	R	S	S	S	S	R	R	S	S	S	S	S	R	S	S	S
++	R	R	n.d.	S	S	R	R	R	S	S	S	n.d.	S	n.d.	S	S	I
--	R	R	S	S	S	R	R	R	S	S	S	S	S	S	S	S	S
++	R	R	S	S	S	S	R	R	S	S	S	S	S	R	S	S	S
--	R	R	S	S	S	S	R	R	S	S	S	S	S	S	S	S	S
--	R	R	S	S	S	R	R	R	S	S	S	S	S	R	S	S	I
--	R	R	S	S	S	S	R	R	S	S	S	S	S	R	S	S	S
+	R	R	S	S	S	S	R	R	S	S	S	S	S	n.d.	S	S	S
--	R	R	S	S	S	S	R	R	S	S	S	S	S	S	S	S	I
+	R	R	S	S	S	S	R	R	S	S	S	S	S	R	S	S	I

Supplementary Figure S1. Neighbor-joining dendrogram resulting from ERIC-PCR for Serratia marcescens clinical isolates (SM01-20), susceptibility to phage SALSA and antibiotic resistance profiles. AMP: ampicillin, SAM: ampicillin/sulbactam, PIP: piperacillin, TZP: piperacillin/sulbactam, CTX: cefotaxim, CPD: cefpodoxim, CXM: cefuroxim, CAZ: ceftazidim, IMP: imipenem, MPM: meropenem, ETP: Ertapenem, CIP: ciprofloxacin; MXF: moxifloxacin, Gen: Gentamicin; SXT: trimethoprim/sulfamethoxazole; TGC: tigecycline; R = resistant, I = intermediate, S = sensitive, n.d.: not determined; ++ = Productive lysis by phage SALSA; + = Lysis from without; - = strain not susceptible to phage SALSA
