# Supplementary material for: Tackling Intrinsic Antibiotic Resistance in Serratia marcescens with a Combination of Ampicillin/Sulbactam and Phage SALSA
Source: Antibiotics (Basel). 2020 Jul 1;9(7):371. doi: 10.3390/antibiotics9070371 (PMC7400198; doi:10.3390/antibiotics9070371)
Supplement: Supplementary file 1 [file antibiotics-09-00371-s001.zip › antibiotics-847709-supple-/FigureS4SM14SAM.pdf]

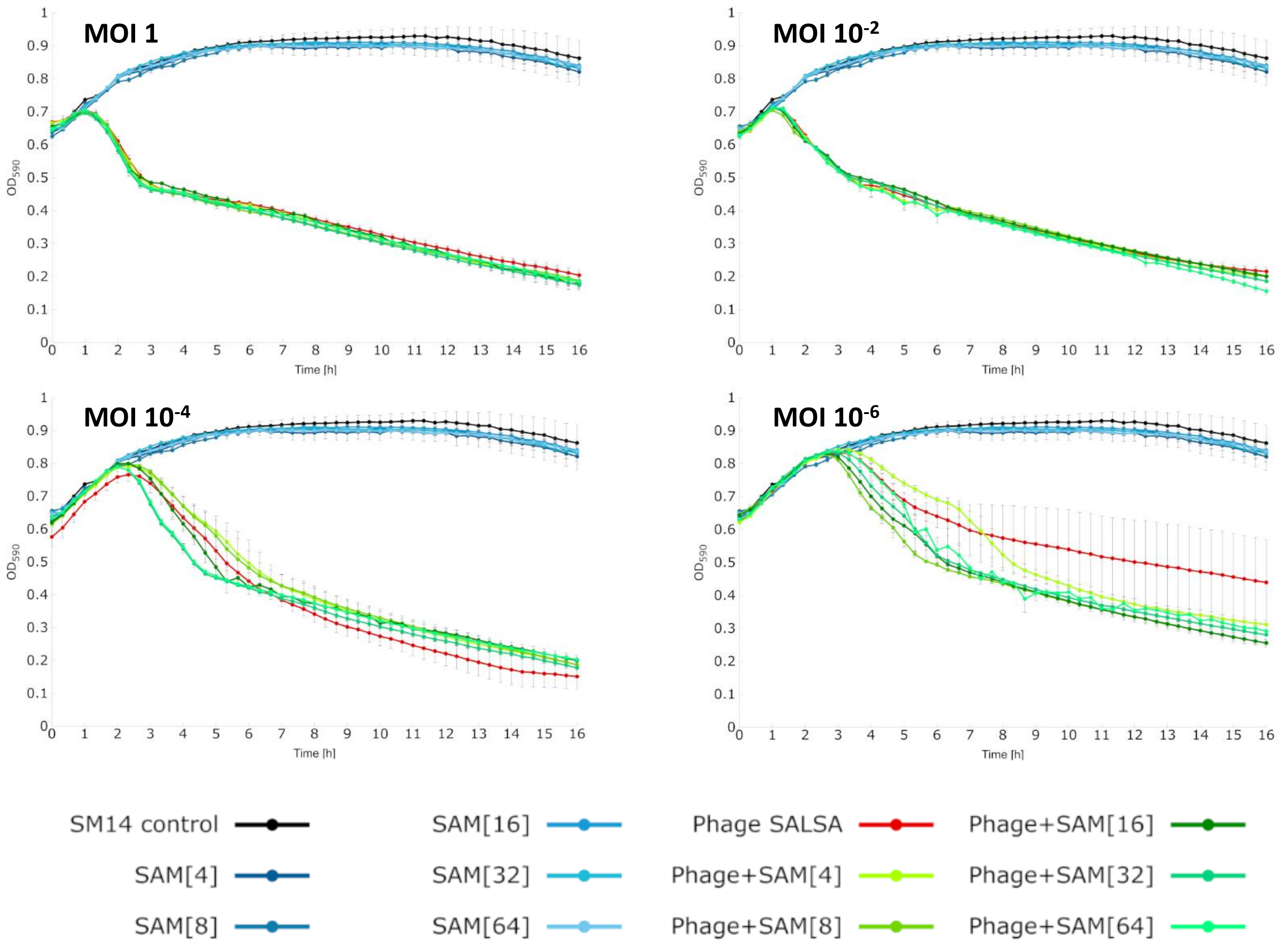

Supplementary Figure S4. Liquid infection assays against *S. marcescens* strain SM14 with or without various concentrations [mg/l] of the antibiotic ampicillin/sulbactam (SAM). Reduction was measured via optical density at 590 nm (OD_{590}). Each experiment was performed in triplicate and the means ± standard errors are indicated.
